# Prevalence of Hepatitis C in the Emilia-Romagna Region of Italy: Population-Wide Screening

**DOI:** 10.3390/v17060843

**Published:** 2025-06-12

**Authors:** Gianmarco Imperiali, Matteo Fiore, Alessandro Bianconi, Giovanna Mattei, Giulio Matteo, Giuseppe Diegoli, Esther Rita De Gioia, Cecilia Acuti Martellucci, Maria Elena Flacco, Lamberto Manzoli, Regional HCV Working Group

**Affiliations:** 1 Section of Hygiene and Preventive Medicine, University of Bologna, 40126 Bologna, Italy; gianmarco.imperiali2@studio.unibo.it (G.I.); matteo.fiore7@studio.unibo.it (M.F.); alessandro.bianconi4@studio.unibo.it (A.B.); estherrita.degioia@studio.unibo.it (E.R.D.G.); c.acutimartellucci@unibo.it (C.A.M.); 2 Emilia-Romagna Region Collective Prevention and Public Health Department, 40127 Bologna, Italy; giovanna.mattei@regione.emilia-romagna.it (G.M.); giulio.matteo@regione.emilia-romagna.it (G.M.); giuseppe.diegoli@regione.emilia-romagna.it (G.D.); 3 Department of Environmental and Prevention Sciences, University of Ferrara, 44121 Ferrara, Italy; mariaelena.flacco@unife.it

**Keywords:** Hepatitis C Virus, screening, HCV prevalence, prison inmates, drug addiction, 1969–1989, Italy

## Abstract

In agreement with WHO recommendations, the Emilia-Romagna Region, Italy, implemented a population-wide HCV screening program for the treatment of the large asymptomatic infected population. From January 2022, the free-of-charge screening targeted all residents born between 1969 and 1989, prison inmates, and injection drug users. Participants were recruited using phone messages, electronic health record notifications, public advertisement, and direct contact with general practitioners. A single blood sample was collected for anti-HCV IgG testing and, if positive, for reflex HCV–RNA testing. Infected subjects were offered an evidence-based therapeutic pathway. By June 2024, 72.8% of high-risk subjects (*n* = 19,732), and 36.9% of the general population (*n* = 488,065) had been screened. A total of 1032 individuals were positive based on the HCV–RNA test, and the detection rate widely differed between the high-risk and the general population (23.8‰ vs. 1.2‰, respectively). Of the infected individuals, 88.1% were seen by a specialist physician, and 74.3% (*n* = 767) started antiviral therapy. Thanks to multiple recruitment approaches, over one third of the general population participated in HCV screening. The program performance was substantially greater among high-risk individuals compared to the general population. To achieve WHO targets, policymakers might consider expanding the screening to other high-risk subgroups and/or adapting birth cohorts.

## 1. Introduction

Hepatitis C Virus (HCV) infection is one of the most prevalent blood-borne disease globally and is a major cause of chronic hepatitis [1,2]. More than 70 million patients are estimated to live with chronic HCV infection world-wide, and approximately 350,000 related deaths occur annually [2,3,4]. If not treated, chronic hepatitis progresses to liver cirrhosis and hepatocellular carcinoma within 20 years in about 10% and 2% of infected patients, respectively [5]. The advent of direct-acting antiviral agents (DAAs) in 2014 drastically improved HCV treatment by offering a simplified protocol and sustained viral response in 95% of patients [6,7]. To address this burden, the World Health Organization (WHO) developed strategies in 2016 to eliminate HCV as a public health threat by 2030, setting the target of reducing its global mortality by 65% [8]. To achieve this goal, countries were encouraged to develop screening programs to increase testing and treatment uptake [1], in order to identify the large population with asymptomatic HCV [7].

Italy has the highest estimated seroprevalence of HCV in Europe and, in line with WHO objectives, has allocated over EUR 70 million to develop free regional screening programs [9,10]. In January 2022, the Emilia-Romagna Region, located in northern Italy, started a population-wide screening program to identify HCV-positive adults and promote treatment [11]. We present the results of the first two years of the screening program, in terms of screening adherence, detection rates, and treatment uptake.

## 2. Materials and Methods

We analyzed several measures of effectiveness of the HCV screening program “C devi pensare” in the Emilia-Romagna Region, from its beginning to June 2024 [12]. The program targeted all residents (or those temporarily living in the region) born between 1969 and 1989, plus prison inmates and individuals with drug addiction, regardless of their official residency or age, although data were only collected for individuals aged 18 years or over). The rationale for limiting invitations to the 1969–1989 birth cohorts lies in the estimated highest prevalence of HCV infection and the proportion of immigrants [12,13], and in the prospective advantage conferred by DAA treatment in this age group [14]. The subjects from the eligible birth cohorts were recruited via automated short message service (SMS) and notifications on their electronic health record (EHR), and individuals under institutional care (prison inmates and individuals with drug addiction disorders) were actively recruited by their healthcare providers. The people who injected drugs were identified among the users of outpatient services at National Health Service addiction recovery clinics [15]. Also, general practitioners within the National Health Service were asked to invite their patients to participate, informing them about the purpose and procedures and the therapeutic pathway in the case of a positive result. Finally, screening was offered to eligible individuals when scheduling blood tests at public healthcare facilities. The program also employed various communication strategies to encourage participation, including billboard advertisements in public spaces, a social media campaign, and the distribution of multilingual flyers to reach immigrant communities [11].

All public hospitals and outpatient blood collection facilities located in Emilia-Romagna were required to participate. After written informed consent was obtained, a single venous blood draw was collected, stored, and sent for HCV testing at a single Microbiology laboratory, designated by each Local Health Unit (LHU). No prescription from the primary care physician was requested, and the exam was free of charge. Each blood sample underwent a two-step reflex testing pathway: it was screened for anti-HCV IgG antibodies by enzyme-linked immunosorbent assay (ELISA); if reactive, it was further evaluated for HCV-RNA detection using real-time polymerase chain reaction (RT-PCR) technology.

The subjects who also tested positive in the second test were diagnosed with a confirmed active infection and promptly contacted by a specialist physician, who explained the therapeutic pathway and proposed the start of therapy according to the Italian national guidelines [16]. The physicians were specialists in Infectious Diseases or Gastroenterology who worked in the reference center of each LHU. The test results were uploaded to web-based platforms and sent to LHU data centers. After referral to an healthcare center for further care, the screening data were anonymized in compliance with European General Data Protection Regulation (GDPR) policies [17]. Only aggregated data were then shared with the Emilia-Romagna Region Welfare Department for the current analyses. As the study was conducted using only aggregated, already collected data, tailored to an evaluation of efficacy explicitly requested by the Italian Minister of Health, the Emilia-Romagna Region approved the analysis protocol internally and did not require a formal evaluation from the Regional Ethics Committee.

The outcomes of the study were the following:

- Anti-HCV IgG Screening Adherence—Proportion of individuals who underwent the first-level test among all the invited subjects;
- HCV-RNA Screening Adherence—Proportion of individuals who underwent the second-level test among the subjects who tested positive in the first-level test (anti-HCV IgG);
- Detection Rate—Proportion of individuals who tested positive in the confirmatory HCV-RNA test among the subjects who underwent the first-level test (anti-HCV IgG test);
- Positive Predictive Value (PPV) of the Anti-HCV IgG Screening Test—Proportion of individuals who tested positive in the confirmatory HCV-RNA test among the subjects that tested positive in the first-level test (conventionally calculated for screening programs to assess the performance of first-level tests in correctly identifying, out of an asymptomatic population, the individuals at high risk of having the disease [18,19,20]);
- Outpatient Visit Adherence—Proportion of individuals who were visited at least once by a specialist physician among the subjects that tested positive in the confirmatory HCV-RNA test;
- Treatment Uptake—Proportion of individuals who started antiviral therapy among the subjects who tested positive in the confirmatory HCV-RNA test.

Descriptive statistics were used to summarize the main characteristics of the sample and compute the primary outcomes. For HCV infection detection rate, the specialist visit adherence rate, and the antiviral therapy uptake, 95% confidence intervals (CIs) were computed using Stata, version 13.0 (Stata Corp., College Station, TX, USA, 2013).

## 3. Results

Overall, 1,364,341 individuals were eligible for the HCV screening program from January 2022 to June 2024: 1,330,562 were residents born between 1969 and 1989; 24,678 were individuals with drug addiction disorders, and 9101 were prison inmates (Table 1). Almost all the subjects of the birth cohort (99.4%), and approximately 80% of the individuals in institutional care were invited to participate.

The adherence to the first-level test largely varied across subgroups: the majority of the invited prison inmates and addiction service users participated to the screening (93.8% and 64.7%, respectively), while the overall uptake in residents in the birth cohort was 36.9%.

The total number of individuals who tested positive in the first-level test (anti-HCV IgG) was 5858, and the positivity rate also differed widely by population group, being much higher among the institutionalized individuals (approximately 100 × 1000 participants) than the residents (8.0 × 1000).

The vast majority of the positive subjects agreed to participate in the second-level screening (approximately 90%; *n* = 5295). Of them, 1032 (19.5%) also tested positive in the second-level, confirmatory HCV-RNA test and were considered infected, corresponding to an overall detection rate of 2.0 × 1000 participants (95% CI: 1.9–2.2). It is important to note that the incidence of infection varied widely across groups, being much higher among the institutionalized subjects (>20 × 1000 participants; *p* < 0.001).

**Figure 1:**
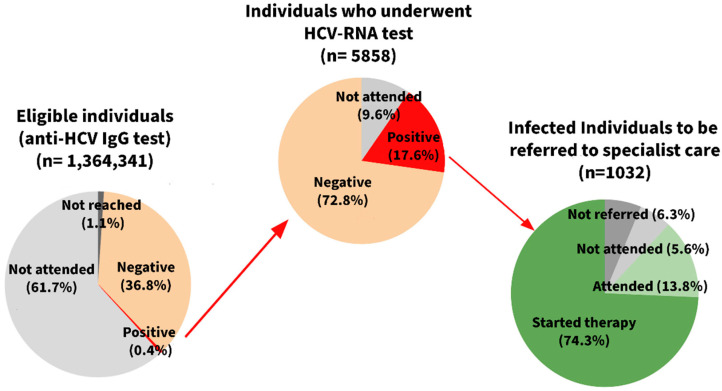
Pathway of the Hepatitis C Virus (HCV) infection screening program in the Emilia-Romagna Region (data as of 30 June 2024).

With regard to program performance (Figure 1 and Table 1), the overall positive predictive value of the first-level test again differed largely by population, with lower values observed among the residents (15.5%) than the other subgroups (28.3%). In contrast, although a high adherence to the treatment program was observed in all groups, the residents showed a higher participation in both the outpatient visit and drug therapy (95.9% and 80.4%, respectively), especially when compared to the addiction service users (75.8% and 63.9%, respectively).

## 4. Discussion

The Emilia-Romagna Region carried out the first Western European study reporting the results of HCV screening in both the general population (birth cohorts 1969–1989) and underserved subgroups, such as prison inmates and individuals with drug addiction disorders. The main findings from the first two years of this population-wide campaign are the following: (a) it was possible to identify a total of 1032 HCV chronically infected subjects, 88.1% of whom were enrolled in the designated clinical pathways; (b) the overall screening performance (screening adherence, detection rate, and positive predictive value) was substantially greater among the institutionalized subjects (prison inmates and persons with drug addiction disorders) than in the general population, highlighting the need to enroll these hard-to-reach segments of population.

The overall adherence to HCV screening in the general population was approximately 40%. On one hand, a few similar programs in other countries have reached higher levels [21]. On the other hand, many programs have shown worse results, and the only other screening (against colorectal cancer) targeting the general population of both sexes in the same region showed a very similar 40% adherence, although it was activated two decades prior [22,23]. The fact that the residents had already used screening services for a long time might have contributed to the relatively high adherence to a newly established screening system against an infectious disease. Indeed, the Emilia-Romagna region started in the 1990s to provide free, organized screening programs for colon, breast, and cervical cancers [24,25].

Thus far, two large population studies have reported on the effectiveness of DAAs in chronically HCV-infected patients: a sharp decrease in all-cause mortality and hepatocellular carcinoma incidence was observed in a French cohort study, while a decline in liver-related mortality and decompensated cirrhosis diagnoses, together with a plateau in the previously increasing rates of hepatocellular carcinoma and all-cause mortality, were documented in an Australian pre-post evaluation [26,27]. In line with these findings, in this program, the early identification of more than a thousand HCV-infected subjects, which lead to the subsequent treatment of more than 700 patients, represented a step forward toward the WHO’s 2030 HCV elimination targets [8]. To fully achieve these targets, however, several authors suggested extending the screening program to an older population [28,29,30,31,32]. Clearly, such an extension should be informed by an in-depth cost–benefit analysis, which requires data on HCV prevalence, diagnostic test accuracy, DAA therapy effectiveness, and cost [14,33,34]. This information is not available yet, as this is the first interim report, and a comprehensive evaluation will be performed five years after the start of the screening.

The HCV-RNA detection rate recorded in the general population of the Emilia-Romagna was comparable to the one recently reported from the 1969–1989 birth cohort in the neighboring Lombardy region (1.0‰) [10]. Similarly, two Italian screenings in the same birth cohorts recently reported detection rates of 0.5‰ in a COVID-19 vaccination setting and 0.7‰ among hospitalized patients [35,36]. In line with a recent modeling study by the Polaris Observatory [32], these findings suggest that the Italian HCV chronic infection prevalence is similar to rates in other Western European countries (3.0‰) and significantly lower than the values typically reported in Central and Eastern European nations (8.0‰ and 29.0‰, respectively). Indeed, in 2016, Italy was thought to have the highest HCV seroprevalence in the EU [9]. In contrast, this study showed an anti-HCV IgG detection rate comparable to other European countries (5.4‰ to 15.0‰, among the general population) [37]. This considerably lower seroprevalence may be explained by three main factors: (a) the fact that previous estimates relied on studies published nearly twenty years ago that included populations from Southern Italy, where the prevalence is higher; (b) the cumulative effect of time (aging, all-cause and liver-related deaths, and declining incident infections); and (c) the growing use of effective treatments [38,39,40]. When the results of additional government funded region-wide screening programs are available, it will be possible to more precisely update the Italian HCV national burden estimates.

As mentioned, the individuals under institutional care exhibited much higher HCV-RNA detection rates than the general population, in agreement with the well-established evidence regarding HCV prevalence among high-risk populations and transmission patterns (e.g., injection drug use) [41,42]. In settings characterized by high financial constraints, this finding confirms the importance of implementing targeted policies and defined micro-elimination strategies to identify and cure HCV-infected individuals among high-risk populations with a greater cost-effective approach [43]. The adherence rate to the first-level test was also higher among the underserved subjects compared to the general population, suggesting that the tailored recruitment strategies had a positive impact in engaging such hard-to-reach subgroups [41,42,44,45,46]. Indeed, while the adherence rate to the first-level test in the general population was not substantially higher than those reported in other HCV screening studies [10,21], the screening uptake among underserved subgroups was higher compared to most of the recent literature [47,48,49,50,51,52], probably because many of these individuals were already being screened in the context of their care pathways. Overall, given the above findings, HCV screening could be effectively extended to additional subgroups that are known to have a higher prevalence of HCV chronic infection compared to the general population (e.g., patients undergoing hemodialysis, sex workers, and men who have sex with men—MSM) [47]. Indeed, MSM were not targeted by the Emilia-Romagna screening program, although an HCV chronic infection prevalence of 3.8‰, estimated by a recent meta-analysis, suggests that the early diagnosis of HCV infection could prevent substantial morbidity in this group [53].

Consistent with several previous studies [36,54,55,56], reporting rates of true-positive subjects ranging from 18% and 36% after the first-level test; in this sample, we found that approximately one out of five subjects with a positive anti-HCV IgG test actually tested positive in the HCV-RNA test. As suggested by Beltrami et al. [57], this could be partially explained by the absence of exclusion criteria in the screening program, leading to the inclusion of individuals already treated with DAAs or on regular follow-up for known HCV infection. Future research is needed to estimate the impact of potential exclusion criteria on the PPV of the first-level test, which would allow us to refine the screening protocol and reduce the false-positive rate.

Despite employing a centralized, validated one-step method to perform two laboratory tests together, from a single blood draw, approximately 10% of the samples could not be assessed for the confirmatory test (HCV-RNA detection). It is worth noting that this loss of data was not due to laboratory, management, or informatics deficiencies but was caused by directives issued by the Data Protection Authority of one LHU. In this unit, at the beginning of the program, the second-level test was not allowed due to concerns regarding informed consent. The format of the informed consent was rapidly revised, and the limitation was resolved within the first 15 days of the program, but we could not process the samples collected before the change.

In this program, almost nine out of ten HCV-infected individuals began the specialized care pathway provided by the program. This robust linkage-to-care result represents an important accomplishment, given the heterogenous performances recorded in the literature [42,45,58,59]. However, for once, this indicator of screening performance was poorer among the individuals in institutional care compared to the general population, suggesting the existence of potential barriers in these subgroups, such as the high turnover rate and frequent transfers among prisoners, and socio-economic vulnerabilities among subjects with drug abuse disorders [42,45,46]. A recent systematic review suggests potential strategies to enhance the drug abuser linkage-to-care result, including individual navigation and education regarding the tolerability of DAA therapy [41].

The strengths of this study include the involvement of all the residents of the Emilia-Romagna Region (born between 1969 and 1989), prison inmates, and individuals with drug addiction disorders, with a tailored pathway for these high-risk, hard-to-reach subgroups and the use of a reflex test, requiring a single blood draw, processed with a validated two-step technology. However, this study also has limitations that must be considered when interpreting the results. First, as the study design was based on aggregated data, because of strict privacy requirements, the demographic and clinical characteristics of the subjects who participated in the program could not be analyzed. Second, as the individuals who tested negative did not undergo the confirmatory test, it was not possible to assess the specificity and sensitivity of the first-level test. Third, given that the screening was directed towards the entire population, some of the positive subjects already knew that they were infected. This helped in producing more reliable estimates of the infection prevalence but may have reduced the benefit to the population, as some of the individuals were already on therapy and the screening was only useful as a monitoring tool. Finally, as mentioned, we could not evaluate the effectiveness of the therapy and the costs of the program at this stage.

In conclusion, the HCV screening program in the Emilia-Romagna Region, conducted from January 2022 to June 2024, targeting both the general population (1969–89 birth cohort) and individuals under institutional care, identified 1032 new chronic infections, the vast majority of which have been taken care of by a specialist physician. Thanks to multiple recruitment approaches, more than one third of the general population participated to the program. The screening adherence, detection rate, and positive predictive value were substantially greater among prison inmates and subjects with drug addiction disorders compared to the general population, thanks to tailored strategies engaging these hard-to-reach subgroups, highlighting the value of these approaches. Policymakers might consider expanding screening to other high-risk subgroups and adapting the birth cohorts targeted as new data emerge to achieve the HCV elimination targets recommended by the WHO. As the program remains active, future analyses will focus on the long-term cost–benefit ratio of the program.

## Figures and Tables

**Table 1:** Results of the HCV infection screening program in the Emilia-Romagna Region, overall and by population subgroup (data as of 30 June 2024).

Screening Outcomes	Overall	Birth Cohort 1969–89	Individuals with Drug Addiction Disorders	Prison Inmates
Eligible individuals, *n*	1,364,341	1,330,562	24,678	9101
Invited individuals, *n*	1,350,032	1,322,915	19,593	7524
Anti-HCV IgG test				
Attended the first-level test, *n*	507,797	488,065	12,671	7061
Screening adherence, % ^A^	37.6	36.9	64.7	93.8
Tested positive, *n*	5858	3911	1271	676
Anti-HCV IgG test positivity rate, ‰ ^B^	11.5	8.0	100	95.7
HCV-RNA confirmatory test				
Attended the second-level test, *n* ^C^	5295	3636	1140	519
Tested positive	1032	562	310	160
HCV-infection detection rate, ‰ (95%CI) ^D^	2.0 (1.9–2.2)	1.2 (1.1–1.3)	24.5 (21.8–27.3)	22.7 (19.3–26.4)
Program performance				
PPV of anti-HCV IgG test ^E^	19.5	15.5	27.2	30.8
Infected subjects who were visited at least once by a specialist physician	909	539	235	135
Outpatient visit adherence, % (95%CI) ^F^	88.1 (85.9–90.0)	95.9 (93.9–97.4)	75.8 (70.6–80.5)	84.4 (77.8–89.6)
Infected subjects who started antiviral therapy	767	452	198	117
Drug therapy uptake, % (95%CI) ^G^	74.3 (71.5–77.0)	80.4 (76.9–83.6)	63.9 (58.2–69.2)	73.1 (65.6–79.8)

HCV, Hepatitis C Virus; IgG, Immunoglobulin G; CI, confidence interval. ^A^ Proportion of individuals who underwent the first-level test (anti-HCV IgG test) among all invited subjects. ^B^ Proportion of individuals who tested positive in the first-level test (anti-HCV IgG), rate ×1000. ^C^ Number of individuals who underwent the second-level test among the subjects who tested positive in the first-level test (anti-HCV IgG test): this corresponded to an overall proportion of 90.4%, and to 93.0%, 89.7%, and 76.8%, respectively, among the general population, individuals with drug addiction disorders, and prison inmates. ^D^ Individuals who tested positive in the confirmatory HCV-RNA test among the subjects who underwent the first-level test (anti-HCV IgG test), rate ×1000. ^E^ Positive Predictive Value: proportion of individuals who tested positive in the confirmatory HCV-RNA test among the subjects who tested positive in the first-level test (anti-HCV IgG test). ^F^ Proportion of individuals who were visited at least once by a specialist physician among the subjects who tested positive in the confirmatory HCV-RNA test. ^G^ Proportion of individuals who started antiviral therapy among the subjects who tested positive in the confirmatory HCV-RNA test.

## Data Availability

The original contributions presented in this study are included in the article. Further inquiries can be directed to the corresponding author.
